# Vitamin A Intake and Risk of Cancer Incidence: Insights from a Case–Control Study

**DOI:** 10.3390/nu17172744

**Published:** 2025-08-25

**Authors:** Shunya Ikeda, Ngoc Bao Truong, Anh Hue Tran, Thinh Gia Nguyen, Lam Tung Luong, Linh Thuy Le, Ngoan Tran Le

**Affiliations:** 1 Canon Institute for Global Studies, Tokyo 100-6511, Japan; 2 School of Medicine, International University of Health and Welfare, Narita City 286-8686, Japan; 3 Laboratory of Embryology and Genetics of Human Malformation, Institut National de la Santé et de la Recherche Médicale, Unité Mixte de Recherche (INSERM UMR), Imagine Institute, 75015 Paris, France; 4 Institute of Research and Development, Duy Tan University, Da Nang City 550000, Vietnam

**Keywords:** cancer risk, U-shaped, vitamin A intake, observational study

## Abstract

**Background**: The association between dietary vitamin A intake and cancer risk remains unclear. There may be under-researched links between dietary vitamin A and cancer. This study aimed to clarify this relationship and a possible reference vitamin A intake. **Methods**: We conducted a hospital-based case–control study. Exposure data was determined from participants, including 3758 incident cancer cases (esophagus, stomach, colon, rectum, lung, breast, and other cancers) and 2995 hospital controls before the day of surgery treatment at the same hospitals. Dietary vitamin A intake was assessed using a validated semi-quantitative food frequency questionnaire. Odds ratios (ORs) and 95% confidence intervals (CIs) were calculated to evaluate the association between vitamin A intake and cancer risk. Restricted cubic splines suggest a safe range of vitamin A intake of 85.3–104.0 µg/day, which is a reference quantile. **Results**: We found a U-shaped association between vitamin A intake and cancer incidence compared to the reference. Both the lowest and highest intakes were associated with an increased cancer risk, with OR (95% CI) values 1.98 (1.57, 2.49) and 2.06 (1.66, 2.56), respectively. This U-shaped pattern was consistent across subgroups defined by sex, body mass index, smoking status, alcohol consumption, blood type A, and cancers of the esophagus, stomach, breast, and rectum, but not lung and colon cancer. The U-shaped relationship remained after adjusting for dietary vitamin A intake per kg of body weight and vitamin A–energy residual estimation adjustment. Confidence intervals were wider at the highest exposure levels. **Conclusions**: We observed a U-shaped relationship between vitamin A intake and the risk of cancer incidence, with a reference dietary vitamin A intake of 85.3–104.0 µg/day. These findings warrant further investigation to understand the mechanisms of this U-shaped association.

## 1. Introduction

Vitamin A plays a critical role in various physiological processes, including vision, immune function, cell differentiation, and embryonic development. Some carotenoids with provitamin A activity function as antioxidants, reducing oxidative stress and inflammation, which may contribute to the prevention of chronic diseases, including cancer [1].

The association between vitamin A intake and cancer is inconsistent, as epidemiological studies and clinical trials have provided different results [2,3,4]. A meta-analysis has concluded that high dietary consumption of vitamin A decreases the incidence of breast and ovarian cancers [5]. On the other hand, clinical trials have shown that vitamin A increases the risk of death from lung and prostate cancer [4]. A U-shaped association between plasma retinol and the risk of all-cause mortality was noted [6]. Another study suggested a possible U-shaped association between plasma retinol levels and non-digestive system cancers [7].

Cancer is a major global health burden, with approximately 20 million new cases and 9.7 million deaths reported worldwide [8], including in Vietnam, where cancer incidence and mortality are also high, underscoring the need for preventive strategies [9]. Because there is possibly a U-shaped association between this vitamin and cancer risk, and potentially a reference dietary intake level of vitamin A, we examined the association between dietary vitamin A intake and cancer risk in a large case–control study in Northern Vietnam.

## 2. Methods

This research project was supported by the Ministry of Science and Technology of Vietnam and the Japanese government from 2003 to 2019. Results from the project were previously presented [10,11].

### 2.1. Study Design and Setting

We conducted a hospital-based case–control study. Participants were recruited weekly from four major university hospitals in Hanoi, Vietnam. All cancer diagnoses were confirmed through pathology. The study protocol was approved by the scientific councils of the participating hospitals, allowing direct patient contact, interviews, and access to medical records. Detailed sources of data have been previously published [10,11].

### 2.2. Recruitment of Cancer Patients

A total of 3758 incident cancer patients were recruited, including cases of esophageal (*n* = 195), stomach (*n* = 1182), colon (*n* = 567), rectal (*n* = 482), lung (*n* = 225), breast (*n* = 281), and other cancers (*n* = 826). Trained interviewers interviewed the selected incident cancer patients before the surgical treatment. The inclusion criteria were informed consent and a confirmed pathological diagnosis before surgery. The exclusion criteria included refusal to participate in the study or a poor general health condition. The selection criteria for incident cancer cases were implemented as previously described [10,11].

### 2.3. Recruitment of Hospital-Based Controls and Matching

The study aimed for a 1:1 case-to-control ratio. Control participants were matched to cases by sex and within five-year age groups. However, cancer patients were hospitalized in much larger numbers than non-cancer patients during the study period. Therefore, 3758 cancer patients were recruited, with only 2995 hospital controls. Controls were patients newly admitted to the hospital for surgery due to non-cancerous conditions such as trauma, urinary tract stones, cholelithiasis, hernias, hyperhidrosis, kidney donation, benign prostatic hyperplasia, hemorrhoids, thyroid nodules, and other non-cancer diagnoses. The trained interviewers interviewed the selected hospital controls before their surgical treatment. The inclusion criteria for controls included agreement to participate in the study and not suffering from any cancer. The exclusion criteria included refusal to participate or a poor general health condition. The selection criteria for incident control cases were implemented as previously described [10,11].

### 2.4. Development of a Semi-Quantitative Food Frequency Questionnaire

The semi-quantitative food frequency questionnaire (SQFFQ) was initially designed in 2003 based on a household nutritional survey using 24 h dietary record methods [12]. By similar methods, it was updated and revised in 2017 to select food items for the study. The 2003 version of the SQFFQ included 80 food items, and the 2017 version included 85. Both focused on unprocessed food, including locally available fresh whole foods and natural, local farm products (e.g., liver, eggs, fresh dark leafy greens, vegetables, and colorful fruits). Consumption frequencies were categorized into seven levels, ranging from 1 to 3 times per day to never in the past year. The SQFFQ was developed as previously described [12,13].

### 2.5. Validation of SQFFQ

The validity of the SQFFQ was evaluated against 24 h dietary recalls in a sample of 300 households (1327 individuals). Correlation coefficient (*R*^2^) for energy-adjusted nutrient intake ranged from 0.20 (lipids) to 0.53 (energy intake), with protein at 0.38 [12,13]. Reproducibility was additionally assessed in 150 healthy adults who completed the SQFFQ twice, 2–3 weeks apart, by independent interviewers. The test–retest correlation coefficient (*R*-squared) was 0.38 for vitamin A intake, 0.65 for protein, 0.44 for lipids, and 0.84 for energy. For energy adjustment, we applied the residual model [14], regressing vitamin A intake on total energy intake and analyzing the residuals. The distribution of residuals was assessed using Kernel density estimation, indicating that the residuals were more likely to be normally distributed (Supplementary Figure S1).

### 2.6. Assessment of Vitamin A Intake

Dietary vitamin A intake was calculated using the 2019 Vietnam Nutritional Composition Table. Daily food consumption in grams and frequency data from the SQFFQ were used to estimate daily vitamin A intake. Full details of the estimation procedures have been described in previous publications. Intake estimates were in retinol µg/day in the present study (vitamin A (1 µg) equated to (1 µg) of retinol, overall mean ± SD: 102.6 µg/day ± 93.9 (SD: standard deviation)). Retinol Equivalent (RE, one µg) equates one µg of retinol to 6 µg of dietary β-carotene, 12 µg of dietary α-carotene, and 12 µg of dietary β-cryptoxanthin. Retinol Activity Equivalent (RAE, one µg) is equivalent to 1 µg of retinol, 12 µg of dietary β-carotene, 24 µg of dietary α-carotene, and 24 µg of dietary β-cryptoxanthin [15].

### 2.7. Covariates

Information on potential confounders was collected, including sex, age (15–39, 40–49, 50–59, 60–69, 70–79, ≥80), education level (primary, secondary, high school or higher), blood type (A, B, AB, O), body mass index (BMI, kg/m^2^, classified as <18.5, 18.5–<23, 23–<25, ≥25), alcohol consumption (yes/no), smoking status (ever/never), coffee consumption (yes/no), diabetes history (yes/no), family history of cancer (yes/no), total energy intake (kcal/day, in nine quantiles), and data collection period. These covariates were considered for adjustment in multivariable models based on their potential association with both vitamin A intake and cancer risk [10,11,16].

### 2.8. Statistical Analysis

**Figure 1:**
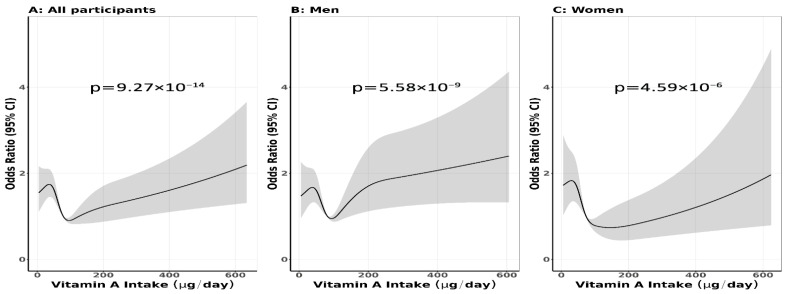
Restricted cubic splines of the association between vitamin A intake and cancer risk. OR (95% CI): Odds ratio and 95% confidence interval (non-linear, all *ps* < 0.001).

Differences in continuous and categorical variables between cases and controls were assessed using t-tests and χ^2^ tests, respectively. The unconditional logistic regression model was used to estimate odds ratios (ORs) and 95% confidence intervals (CIs) for the association between vitamin A intake and cancer risk. Multivariable models were adjusted for the covariates listed in Section 2.7. Model 1 adjusted for sex, age group, highest education level, blood group, BMI, alcohol consumption, family history of cancer, smoking status, history of diabetes, coffee drinking, total energy intake, and four periods of data connections. Model 2 was Model 1 plus vitamin A–energy residual estimation [14]. Stratified analyses were conducted by sex (men and women), smoking status (never and ever smoker), BMI (<23 and ≥23 kg/m^2^), alcohol consumption (never and ever drinker), and blood type (A, AB, B, O), and examined for vitamin A intake per kilogram of body weight. A sensitivity analysis was conducted, excluding cases with early symptoms to avoid changes in food consumption (cancer cases, *n* = 1996; control cases, *n* = 2764) and matching for sex and age within ±5 years (cancer cases, *n* = 1822; control cases, *n* = 1822). Details are mentioned in the Supplementary Materials. Since there is no preconceived notion of the recommended or safe vitamin A intake for the Vietnamese population, the reference range for intake was defined as the quantile nearest to the overall mean and median intake (85.3–104.0 µg/day)—which was chosen based on the curve of restricted cubic splines showing a safe level of vitamin A—and internal distribution, and is consistent with a previous study. *p*-values for trend were calculated by modeling vitamin A intake as a continuous variable (per standard deviation) above and below the reference quantile (85.3–104.0 µg/day). Restricted cubic spline analysis was conducted for the non-linear association between dietary vitamin A intake and cancer risk (Figure 1).

### 2.9. Ethical Considerations

The study was approved by the Institutional Review Board for Ethics in Biomedical Research at Hanoi Medical University (Approval No. 3918/HMUIRB, dated 25 December 2018; 61/HMURB, dated 25 November 2008) and the IRB of the International University of Health and Welfare, Japan (Approval No. 19-Ig-17, dated 27 May 2019). Informed consent was obtained from all participants.

## 3. Results

The percentage of men among cancer patients was higher than that of women (60.3% vs. 39.7%). Cancer patients had a higher smoking rate (41.7% vs. 39.2%), a higher prevalence of blood type A (30.0% vs. 20.8%), and a greater percentage of BMI < 18.5 kg/m^2^ (35.9% vs. 18.6%) compared to the control group. Intake estimates were reported in retinol, µg/day, in the present study, with an overall mean ± SD of 102.6 µg/day ± 93.9 (SD: standard deviation) [15] (Table 1). In addition, we also estimated Retinol Equivalent (RE)— mean ± SD: 627.3 µg/day ± 422.4—and Retinol Activity Equivalent (RAE)—mean ± SD: 364.9 µg/day ± 238.1.

We found a U-shaped association between vitamin A intake and cancer incidence, with a reference dietary vitamin A intake of 85.3–104.0 µg/day. Both the lowest and highest intakes were associated with an increased cancer risk, with OR (95% CI) values of 1.98 (1.57, 2.49) and 2.06 (1.66, 2.56), respectively. A similar U-shaped association persisted when excluding cases with early symptoms (Table 2), and after matching for sex and age within ±5 years (Supplementary Table S1). A U-shaped association was observed in the subgroup analyses, including by sex, the status of body mass index, smoking, alcohol consumption, and blood type (A, B, O) (Supplementary Table S2).

The lowest and highest consumption quantiles were associated with an increased risk of esophagus cancer, with an OR (95% CI) of 1.71 (0.82, 3.56), *p* for trend 0.015, and 2.71 (1.18, 6.22), *p* for trend < 0.001, respectively, and with an increased risk of breast cancer, with an OR (95% CI) of 1.86 (0.86, 4.00), *p* for trend 0.001, and 3.45 (1.36, 8.71), *p* for trend 0.002, respectively (Table 3). The lowest and highest quantile consumptions were also associated with an increased risk of cancer of the rectum and stomach, but not for the colon. The lowest quantile of consumption increased the risk of lung cancer, with OR (95% CI): 2.08 (1.01, 4.30) (Supplementary Table S3).

A similar U-shaped association was observed when vitamin A intake was analyzed per kg of body weight. The lowest and highest quantile consumptions increased cancer risk, with OR (95% CI) values of 1.39 (1.11, 1.75) and 1.82 (1.46, 2.27), respectively. The risk pattern did not change after adjustments for vitamin A–energy residual estimation. The lowest and highest quantile consumption increased cancer risk, with OR (95% CI) values of 2.36 (1.73, 3.20) and 1.62 (1.17, 2.24), respectively (Table 4). Results of restricted cubic spline regression found a U-shaped relationship between dietary vitamin A intake and cancer risk for all participants, and by sex for both men and women, which suggests a safe range of vitamin A intake of 85.3–104.0 µg/day, which is our reference quantile (non-linear, all *ps* < 0.001) (Figure 1). Confidence intervals were wider at the highest exposure levels.

## 4. Discussion

We observed a U-shaped relationship between dietary vitamin A intake and overall cancer risk, with a reference vitamin A intake at 85.3–104.0 µg/day. This association remained consistent across stratified analyses by sex, smoking status, alcohol consumption, body mass index, blood type A, and the specific cancer sites of the esophagus, breast, stomach, and rectum. The U-shaped pattern also persisted after additional adjustments for vitamin A–energy residual estimation and when vitamin A intake was analyzed per kilogram of body weight.

Our findings are based on a large hospital-based case–control study. All cancer diagnoses were pathologically confirmed, and controls were patients recruited from the same hospitals, reducing potential selection bias due to socioeconomic differences. Vitamin A intake was assessed using the validated semi-quantitative food frequency questionnaire. The exposure data was collected before the surgical treatment to minimize the effects of the intervention and the inpatient condition. The consistency of the U-shaped association across subgroups and cancer sites of the esophagus, breast, stomach, and rectum supports the robustness of the findings. When excluding cases with early symptoms to avoid changes in food consumption and matching for sex and age within a ±5-year range, the U-shaped association remained. This suggests that the present findings are reliable.

These results suggest that the reference range of dietary vitamin A intake (85.3–104.0 µg/day) lies close to the population’s median (86.6 µg/day) and mean (108.4 µg/day) intakes. The median dietary intake in this study (78.6 µg/day) was higher than the median intake reported in the 2010 national nutrition survey in Vietnam (40.8 µg/day), indicating that the present estimated vitamin A intake is reliable [17]. The Vietnamese Ministry of Health recommends a daily intake of 600 µg for men and 500 µg for women, using the old unit of “Retinol Equivalent (RE)”. The present estimated RE was 627.3 µg/day ± 422.4; again, the present study’s exposure to vitamin A intake (RE) aligns with the recommendations [18]. Cultural, dietary, and genetic differences between populations may influence vitamin A intake. In Vietnam, vitamin A intake predominantly comes from plant-based sources rich in provitamin A carotenoids. In contrast, in Western countries, preformed vitamin A from animal products and fortified foods is more common [19]. Additionally, polymorphisms in PNLIPRP2, which reduce its enzymatic activity involved in the digestion of dietary retinyl esters, and variants in RBP4 associated with decreased retinol transport, are more prevalent in certain Asian populations [20].

These findings are consistent with previous observational studies, which observed a U-shaped association between vitamin A and cancer or all-cause mortality in prospective cohort studies [6,7,21,22,23,24] and a case–control study [25]. In a meta-analysis, an inverse association was observed between vitamin A intake and cancer risk, with the highest intake group compared to the lowest intake group showing a relative risk (RR) of 0.78 (95% CI, 0.71–0.83). However, among the subgroups of individuals who were given doses at least 4 times above the tolerable upper intake, the RR (95% CI) increased to 1.20 (95% CI, 0.99–1.44). In contrast, for individuals who received doses below the upper intake level, the RR (95% CI) decreased to 0.76 (95% CI, 0.68–0.86). It is also possible that there is a U-shaped association between vitamin A intake and cancer risk [26]. Excess vitamin A intake was associated with an increased risk of cancer in U.S. adults in a cohort study [21]. However, the average intake among U.S. adults in this study was approximately 650 mcg, much higher than the average intake of our study group. The findings suggest that both excessive and insufficient vitamin A intake may increase the risk of cancer.

Because there is consistency between the standard and sensitive analyses, the results may not be a statistical artifact resulting from measurement error, residual confounding, or the specific distribution of intake in this population. The findings are possibly consistent with the biological mechanism and animal studies (Supplementary Materials). Evidence suggests that vitamin A and its derivatives may exert carcinogenic effects under certain conditions. For example, β-carotene has been shown to enhance the activation of carcinogens such as 3-methylcholanthrene and benzo[a]pyrene (B[a]P) [27,28,29]. Moreover, retinoic acid may promote neoplastic proliferation by activating the orphan nuclear receptor PPARβ/δ [27]. The autoxidation of retinoids can also generate hydrogen peroxide (H_2_O_2_), which contributes to DNA damage in the presence of endogenous metals [30]. These biphasic effects of vitamin A have also been observed in animal studies. An experimental study found that vitamin A at an appropriate dose enhanced the antioxidant system in rats; however, the placebo group and the highest dose of vitamin A supplementation showed adverse effects [31]. A very high dose of vitamin A provoked oxidative stress and histopathological liver changes, which supports the biological plausibility of elevated risk at high intake levels [31].

This study has several limitations. First, as with all case–control designs, recall bias is a concern. Second, the population was hospital-based, and the exposure to dietary vitamin A intake might not represent the general population. Lack of serum vitamin A or retinol levels is another significant gap. Additionally, subclinical or latent stages of cancer and early disease symptoms associated with hospital controls may influence nutrient metabolism and intake, possibly distorting dietary data on vitamin A intake [32]. Reverse causation remains a potential limitation, as dietary intake may have been modified in response to subclinical symptoms. Residual confounding from unmeasured variables is also possible despite multivariable adjustments due to the absence of variables related to physical activity, supplement use, and dietary patterns. However, the distribution of residuals was assessed using Kernel density estimation, and it is more likely that the residuals are normally distributed. Since the overall number of controls was fewer than the number of cases, this imbalance between cases and controls may introduce residual confounding factors, as the proportion of individuals with a particular characteristic (e.g., age, sex, smoking, and alcohol consumption) may differ between the two groups [33]. With advanced adjustments, these residual confounding factors due to an imbalance between cases and controls will be minimized. The stratified analyses by cancer site (esophagus, stomach, rectum, and breast) yielded consistent results. Another limitation is that statistical methods to correct for potential bias (e.g., inverse probability weighting or matching) [34] have not yet been examined. The SQFFQ has limited validity for vitamin A (*R*^2^ = 0.38). This substantially reduces confidence in the exposure measurement and may contribute to the observed nonlinearity. Therefore, exposure misclassification may have influenced the results.

Despite these limitations, our study has several notable strengths. It was the first study in Vietnam to explore the role of micronutrients, such as dietary vitamin A intake, in cancer development. This study had a large sample size with multiple cancer sites. Dietary intake was assessed through a face-to-face interview using the validated SQFFQ, which comprises 85 items. In this hospital-based study, controls were within the study hospitals, which helped improve the comparability of our study sample. Multivariable models were used to control for various covariates, thus minimizing the effect of potential confounding factors. Finally, the use of median dietary vitamin A intake as the reference group revealed the relevant intake level for the first time and allowed us to show the U-shaped association between vitamin A intake and cancer risk.

## 5. Conclusions

A U-shaped relationship between the intake level of vitamin A and overall cancer risk was observed in this study. The reference vitamin A intake level ranged from 85.3 to 104.0 µg/day. These findings warrant further investigation to understand the mechanisms of this U-shaped association.

## Figures and Tables

**Table 1:** Characteristics of the study population by case and control.

Characteristics	Controls (*n* = 2995)		Cancer (*n* = 3758)		Total (*n* = 6753)	*p*-Value
	Number	%	Number	%		
Age, mean (SD)	54.5 (12.2)		56.5 (11.9)		55.6 (12.1)	
<40	80	2.7	75	2.0	155	
40–49	287	9.6	262	7.0	549	
50–59	622	20.8	613	16.3	1235	
60–69	916	30.6	1189	31.6	2105	
70–79	781	26.1	1142	30.4	1923	
≥80	309	10.3	477	12.7	786	<0.001
Sex						
Men	1757	58.7	2267	60.3	4024	
Women	1238	41.3	1491	39.7	2729	0.17
Smoking status						
Never smoked	1822	60.8	2190	58.3	4012	
Ever smoked	1173	39.2	1568	41.7	2741	0.033
Alcohol consumption						
Ever drank	1706	57.0	1969	52.4	3675	
Never drank	1289	43.0	1789	47.6	3078	<0.001
Blood type ^a^						
A	477	20.8	838	30.0	1315	
AB	111	4.8	172	6.2	283	
B	671	29.2	758	27.1	1429	
O	1037	45.2	1027	36.7	2064	<0.001
Highest level of education						
Primary school	442	14.8	705	18.8	1147	
Secondary school	1322	44.1	1626	43.3	2948	
High school or higher	1231	41.1	1427	38.0	2658	<0.001
Family history of cancer						
No	2764	92.3	3416	90.9	6180	
Yes	231	7.7	342	9.1	573	0.042
BMI, mean (SD) kg/m^2^	21.3 (3)		20.1 (2.8)		20.7 (2.9)	
<18.5	557	18.6	1348	35.9	1905	
18.5–22.9	1630	54.4	1858	49.4	3488	
23–24.9	509	17.0	391	10.4	900	
≥25	299	10.0	161	4.3	460	<0.001
Coffee drinking status						
Never drank	2289	76.4	3110	82.8	5399	
Ever drank	706	23.6	648	17.2	1354	<0.001
History of diabetes ^a^						
No	2479	93.5	3330	95.7	5809	
Yes	171	6.5	150	4.3	321	<0.001
Total energy intake (kcal/day) mean (SD)	1703.7 (441.4)		1485.5 (506.8)		1582.3 (490.9)	<0.001
Vitamin A intake (µg/day) mean (SD)	108.4 (91.4)		97.9 (95.8)		102.6 (93.9)	<0.001
Median (µg/day)	86.6		71.0		78.6	
Min-max (µg/day)	3.6–655.6		3.6–704.5		3.63–704.45	

^a^ Based on available data; SD is the standard deviation and BMI is the body mass index (Asian category, kg/m^2^). Min-max: minimum–maximum. The Vietnamese Ministry of Health’s recommended daily intake is 600 µg for men and 500 µg for women using the old unit of “Retinol Equivalent (RE)”. Retinol Equivalent (RE, one µg) equates one µg of retinol to 6 µg of dietary β-carotene, 12 µg of dietary α-carotene, and 12 µg of dietary β-cryptoxanthin. Retinol Activity Equivalent (RAE, one µg) is equivalent to 1 µg of retinol, 12 µg of dietary β-carotene, 24 µg of dietary α-carotene, and 24 µg of dietary β-cryptoxanthin [15]. Retinol Equivalent (RE) mean ± SD: 627.3 ± 422.4 µg/day; Retinol Activity Equivalent (RAE) mean ± SD: 364.9 ± 238.1 µg/day.

**Table 2:** Vitamin A intake and total risk of cancer.

Vitamin A Intake, µg/day, Mean (Min–Max)	Control	Cancer	Crude OR (95% CI)	*p*	Adjusted OR (95% CI) ^a^	*p*
Entire study population (cancer cases *n* = 3758, control cases *n* = 2995)						
*p*__Trend_ ^b^			<0.001		<0.001	
18.9 (3.6–29.2)	253	498	2.57 (2.08, 3.16)	<0.001	1.98 (1.57, 2.49)	<0.001
37.2 (29.3–44.3)	257	493	2.50 (2.03, 3.08)	<0.001	1.98 (1.58, 2.48)	<0.001
51.7 (44.4–58.4)	280	470	2.19 (1.78, 2.69)	<0.001	1.68 (1.35, 2.10)	<0.001
64.9 (58.5–71.7)	309	442	1.86 (1.52, 2.29)	<0.001	1.50 (1.21, 1.86)	<0.001
78.5 (71.8–85.2)	353	397	1.47 (1.20, 1.80)	<0.001	1.31 (1.06, 1.61)	0.014
94.1 (85.3–104.0)	425	326	1.00		1.00	
116.3 (104.1–134.3)	379	371	1.28 (1.04, 1.56)	0.019	1.40 (1.13, 1.73)	0.002
155.6 (134.4–178.2)	386	364	1.23 (1.00, 1.51)	0.046	1.44 (1.17, 1.78)	0.001
306.0 (178.3–704.5)	353	397	1.47 (1.20, 1.80)	<0.001	2.06 (1.66, 2.56)	<0.001
*p*__Trend_ ^c^			<0.001		<0.001	
Excluding cases with early symptoms (cancer cases *n* = 1996, control cases *n* = 2764)						
*p*__Trend_ ^b^			<0.001	0.16		
18.3 (3.6–29.2)	241	240	2.40 (1.83, 3.14)	<0.001	1.49 (1.09, 2.04)	0.014
37.1 (29.3–44.3)	242	235	2.33 (1.78, 3.06)	<0.001	1.73 (1.28, 2.35)	<0.001
51.8 (44.4–58.4)	257	197	1.77 (1.35, 2.33)	<0.001	1.53 (1.13, 2.06)	0.006
65.0 (58.5–71.7)	292	212	1.54 (1.18, 2.00)	0.001	1.37 (1.03, 1.83)	0.032
78.5 (71.8–85.2)	316	223	1.60 (1.23, 2.07)	<0.001	1.58 (1.18, 2.09)	0.002
94.3 (85.3–104.0)	391	183	1.00		1.00	
116.2 (104.1–134.3)	337	206	1.41 (1.08, 1.83)	0.011	1.38 (1.03, 1.85)	0.029
155.3 (134.4–178.2)	358	217	1.37 (1.06, 1.77)	0.018	1.43 (1.07, 1.90)	0.015
313.3 (178.3–704.5)	330	283	2.09 (1.62, 2.70)	<0.001	1.98 (1.49, 2.65)	<0.001
*p*__Trend_ ^c^			0.001		0.001	

^a^ Model 1: Adjusted for sex (men and women), age groups (15–39, 40–49, 50–59, 60–69, 70–79, ≥80), highest education level (primary, secondary, high school or higher), blood groups (A, AB, B, O), BMI (kg/m^2^, <18.5, 18.5–<23, 23–<25, ≥25), alcohol consumption (yes/no), family history of cancer (yes/no), smoking status (ever/never), history of diabetes (yes/no), coffee drinking (yes/no), total energy intake (kcal/day, nine quantiles), and four periods of data connections. *p_*_Trend_ ^b^ for the intake below the reference of the range from 85.3–104.0 µg/day, *p_*_Trend_ ^c^ for the intake above the reference of the range from 85.3 to 104.0 µg/day. OR (95% CI): Odds ratio and 95% confidence interval. Min-max: Minimum–maximum.

**Table 3:** Vitamin A intake and risk of specific cancer sites.

Vitamin A Intake µg/day, Mean (Min–Max)	Control	Cancer	Crude OR (95% CI)	*p*	Adjusted OR (95% CI) ^a^	*p*
Esophagus (C15: *n* = 195; control cases *n* = 2995)						
*p*__Trend_ ^b^			0.11		0.015	
17.7 (3.6–29.2)	253	28	3.36 (1.74, 6.50)	<0.001	1.71 (0.82, 3.56)	0.14
37.7 (29.3–44.3)	257	24	2.83 (1.44, 5.58)	0.003	1.62 (0.77, 3.41)	0.20
51.9 (44.4–58.4)	280	33	3.58 (1.88, 6.81)	<0.001	1.98 (0.98, 4.01)	0.06
64.8 (58.5–71.7)	309	24	2.36 (1.20, 4.63)	0.013	1.63 (0.79, 3.38)	0.18
78.6 (71.8–85.2)	353	21	1.81 (0.91, 3.60)	0.09	1.38 (0.66, 2.87)	0.38
94.0 (85.3–104.0)	425	14	1.00		1.00	
116.5 (104.1–134.3)	379	18	1.44 (0.71, 2.94)	0.31	1.94 (0.91, 4.14)	0.08
155.6 (134.4–178.2)	386	20	1.57 (0.78, 3.16)	0.20	2.06 (0.98, 4.34)	0.06
293.6 (178.3–704.5)	353	13	1.12 (0.52, 2.41)	0.77	2.71 (1.18, 6.22)	0.019
*p*__Trend_ ^c^			0.45		<0.001	
Breast cancer, women (C50: *n* = 281; control cases *n* = 1238)						
*p*__Trend_ ^b^			0.023		0.001	
18.4 (3.6–29.2)	126	45	4.51 (2.33, 8.71)	<0.001	1.86 (0.86, 4.00)	0.11
37.8 (29.3–44.3)	103	47	5.76 (2.97, 11.16)	<0.001	3.63 (1.69, 7.77)	0.001
51.9 (44.4–58.4)	130	50	4.85 (2.53, 9.31)	<0.001	2.62 (1.25, 5.50)	0.011
64.87(58.5–71.7)	143	46	4.06 (2.11, 7.81)	<0.001	2.03 (0.96, 4.27)	0.06
78.5 (71.8–85.2)	147	26	2.23 (1.11, 4.50)	0.025	1.56 (0.71, 3.44)	0.26
94.5 (85.3–104.0)	164	13	1.00		1.00	
116.7 (104.1–134.3)	137	19	1.75 (0.83, 3.67)	0.13	2.06 (0.90, 4.74)	0.08
155.1 (134.4–178.2)	158	23	1.84 (0.90, 3.75)	0.09	2.86 (1.28, 6.41)	0.011
285.9 (178.3–704.5)	130	12	1.16 (0.51, 2.64)	0.71	3.45 (1.36, 8.71)	0.009
*p*__Trend_ ^c^			0.25		0.002	

^a^ Model 1: Adjusted for sex (men and women), age groups (15–39, 40–49, 50–59, 60–69, 70–79, ≥80), highest education level (primary, secondary, high school or higher), blood groups (A, AB, B, O), BMI (kg/m^2^, <18.5, 18.5–<23, 23–<25, ≥25), alcohol consumption (yes/no), family history of cancer (yes/no), smoking status (ever/never), history of diabetes (yes/no), coffee drinking (yes/no), total energy intake (kcal/day, nine quantiles), and four periods of data connections. *p_*_Trend_^b^ for the intake below the reference of the range from 85.3 to 104.0 µg/day, *p_*_Trend_ ^c^ for the intake above the reference of the range from 85.3 to 104.0 µg/day. OR (95% CI): Odds ratio and 95% confidence interval. Min–max: Minimum–maximum.

**Table 4:** A sensitive analysis of the association between vitamin A intake and the total risk of cancer.

Control (*n* = 2995)	Cancer (*n* = 3758)	Adjusted OR (95% CI) ^a^	*p*	Control	Cancer	Adjusted OR (95% CI) ^b^	*p*
Vitamin A intake per kg of body weight				vitamin A–energy residual estimation, additionally adjusted			
280	446	1.39 (1.11, 1.75)	0.004	253	498	2.36 (1.73, 3.20)	<0.001
286	440	1.35 (1.08, 1.68)	0.009	257	493	2.29 (1.74, 3.03)	<0.001
287	439	1.34 (1.08, 1.67)	0.009	280	470	1.88 (1.46, 2.42)	<0.001
332	394	1.09 (0.88, 1.35)	0.42	309	442	1.62 (1.29, 2.04)	<0.001
363	363	1.01 (0.82, 1.25)	0.92	353	397	1.35 (1.09, 1.68)	0.006
380	346	1.00		425	326	1.00	
362	364	1.20 (0.97, 1.49)	0.09	379	371	1.31 (1.05, 1.64)	0.017
364	362	1.34 (1.08, 1.66)	0.008	386	364	1.22 (0.93, 1.60)	0.14
321	405	1.82 (1.46, 2.27)	<0.001	353	397	1.62 (1.17, 2.24)	0.003

OR (95% CI): Odds ratio and 95% confidence interval. ^a^ Model 1: Adjusted for sex (men and women), age groups (15–39, 40–49, 50–59, 60–69, 70–79, ≥80), highest education level (primary, secondary, high school or higher), blood groups (A, AB, B, O), BMI (kg/m^2^, <18.5, 18.5–<23, 23–<25, ≥25), alcohol consumption (yes/no), family history of cancer (yes/no), smoking status (ever/never), history of diabetes (yes/no), coffee drinking (yes/no), total energy intake (kcal/day, nine quantiles), and four periods of data connections. ^b^ Model 2: Model 1 and nutrient–vitamin A residual estimation (quintiles).

## Data Availability

The data presented in this study are available on request from the corresponding author. The data are not publicly available due to ethical reasons.

## Supplementary Materials

The following supporting information can be downloaded at: https://www.mdpi.com/article/10.3390/nu17172744/s1. Figure S1: Kernel density estimates for the control cases. Table S1. Vitamin A intake and the risk of cancer, excluding cases with early symptoms and matching for sex and age ± 5 years. Table S2. Vitamin A intake and the risk of cancer by BMI, smoking, alcohol use, blood groups, and sex. Table S3. Vitamin A intake and the risk of specific cancer sites. References [35,36,37,38,39] are cited in the supplementary materials.

## List of Abbreviations

BMI: body mass index; SQFFQ: semi-quantitative food frequency questionnaire; OR (95% CI): odds ratio (95% confidence interval); ICD-10: International Classification of Diseases, Tenth Revision.
