# Supplementary material for: Vitamin A Intake and Risk of Cancer Incidence: Insights from a Case–Control Study
Source: Nutrients. 2025 Aug 25;17(17):2744. doi: 10.3390/nu17172744 (PMC12430326; doi:10.3390/nu17172744)
Supplement: Supplementary file 1 [file nutrients-17-02744-s001.zip › nutrients-3698611-supplementary.pdf]

Vitamin A Intake and Risk of Cancer Incidence: insights from a case-control study

Supplementary documents

Supplementary Methods

Validation of the SQFFQ

The validity of the SQFFQ was evaluated against 24-hour dietary recalls in a sample of 300 households (1327 individuals). Correlation coefficient (R^2) for energy-adjusted nutrient intake ranged from 0.20 (lipids) to 0.53 (energy intake), with protein at 0.38. (1, 2) Reproducibility was additionally assessed in 150 healthy adults who completed the SQFFQ twice, 2-3 weeks apart, by independent interviewers. The test-retest correlation coefficient was R-squared 0.38 for vitamin A intake, 0.65 for protein, 0.44 for lipids, and 0.84 for energy (Data not shown). For energy adjustment, we applied the residual model (3), regressing vitamin A intake on total energy intake and analyzing the residuals. The distribution of residuals was assessed using Kernel density estimation, indicating that the residuals are more likely to be normally distributed. **(Supplementary Figure S1.)**

Statistical Analysis

A sensitivity analysis was conducted, excluding cases with early symptoms to avoid changes in food consumption (Cancer cases, n = 1996; control cases, n = 2764). Matching for sex and age within ±5 years was made (Cancer cases n = 1822, control cases n = 1822). The incident cancer cases were matched with incident control cases among 2,218 men and 1,426 women. A total of 3,644 cases and controls were available for the analysis. A ratio of case-to-control was 1:1.

Supplementary Discussion

The findings are consistent between the standard and sensitive analyses. When excluding cases with early symptoms to avoid changes in food consumption (Cancer cases n = 1996, control cases n = 2764), and matching for sex and age within ±5 years (Cancer cases n = 1822, control cases n = 1822), a U-shaped association remains, between vitamin A intake and cancer incidence. The results might not be a statistical artifact resulting from measurement error, residual confounding, or specific distribution of intake in this population. The findings are possibly consistent with previous studies on the biological mechanism.

The findings are possibly consistent with previous studies on the biological mechanism. Retinoic acid (RA), the biologically active form of vitamin A, modulates gene transcription through two main classes of nuclear receptors: retinoic acid receptors (RARs) (4) and retinoid X receptors (RXRs). (5) These receptors bind to specific DNA sequences called retinoic acid response elements (RAREs) in the promoters of target genes. (4, 5) This interaction leads to the recruitment of co-activators or co-repressors, ultimately influencing the transcription of target genes. The retinoid receptors also mediate the effects of small lipophilic ligands on gene transcription. (5, 6) One of the most potent RARs, the RARβ receptor, is a critical regulator of cell growth inhibition that plays a crucial role in squamous cell carcinoma. Anti-tumor effects of retinoids have also been demonstrated in colorectal cancer through multiple pathways. In cell lines exhibiting β-catenin accumulation or constitutive activation of

the Wnt/β-catenin pathway, retinoid administration was observed to promote β-catenin degradation. In cancer cells lacking PI3K/Akt pathway inhibition, retinoids inhibit PI3K activity and suppress tumor cell metastasis. (7, 8) On the other hand, evidence also suggests that vitamin A and its derivatives may exert carcinogenic effects under certain conditions. For example, β-carotene has been shown to enhance the activation of carcinogens such as 3-methylcholanthrene and benzo[a]pyrene (B[a]P). (9-11) Moreover, retinoic acid may promote neoplastic proliferation by activating the orphan nuclear receptor PPARβ/δ. (9) The autoxidation of retinoids can also generate hydrogen peroxide (H_2O_2), which contributes to DNA damage in the presence of endogenous metals. (12) These biphasic effects of vitamin A have also been observed in animal studies. An experimental study found that vitamin A at an appropriate dose enhanced the antioxidant system in rats; however, the placebo group and the highest dose of vitamin A supplementation showed adverse effects. (13) Additional molecular and epidemiological research is required to understand better the differential effects of vitamin A on each type of cancer.

Supplementary Tables

Supplementary Table S1. Vitamin A intake and the risk of cancer, excluding cases with early symptoms and matching for sex and age ± 5 years,

Vitamin A intake μg/day, mean (min-max)	Control	Cancer	Crude OR (95% CI)	*P*	Adjusted OR (95% CI) [a]	*P*
**Excluding cases with early symptoms and matching for sex and age ± 5 years, (Cancer cases n = 1822, control cases n = 1822)**						
$P_{\text{\_Trend}}$ [b]			0.001		0.29	
18.5 (3.6-29.2)	151	215	2.39 (1.80, 3.17)	<0.001	1.51 (1.08, 2.09)	0.015
36.9 (29.3-44.3)	147	214	2.44 (1.83, 3.25)	<0.001	1.80 (1.31, 2.48)	<0.001
51.8 (44.4-58.4)	169	174	1.73 (1.30, 2.30)	<0.001	1.49 (1.09, 2.05)	0.013
66.1 (58.5-71.7)	206	195	1.59 (1.21, 2.09)	0.001	1.41 (1.04, 1.91)	0.026
78.6 (71.8-85.2)	207	207	1.68 (1.28, 2.20)	<0.001	1.71 (1.27, 2.31)	<0.001
94.4 (85.3-104.0)	275	164	1.00		1.00	
116.1 (104.1-134.3)	227	197	1.46 (1.11, 1.91)	0.007	1.43 (1.06, 1.94)	0.020
155.5 (134.4-178.2)	237	195	1.38 (1.05, 1.81)	0.020	1.47 (1.09, 1.99)	0.011
310.3 (178.3-704.5)	203	261	2.16 (1.65, 2.81)	<0.001	2.11 (1.56, 2.86)	<0.001
$P_{\text{\_Trend}}$ [c]			<0.001		0.001	

[a] Model 1: Adjusted for sex (men and women), age groups (15-39, 40-49, 50-59, 60-69, 70-79, ≥80), highest education level (primary, secondary, high school or higher), blood groups (A, AB, B, O), BMI (kg/m^2, <18.5, 18.5-<23, 23-<25, ≥25), alcohol consumption (yes/no), family history of cancer (yes/no), smoking status (ever/never), history of diabetes (yes/no), coffee drinking (yes/no), total energy intake (kcal/day, nine quantiles), and four periods of data connections. *P_trend* [b] for the intake below the reference of the range from 85.3-104.0 μg/day, *P_trend* [c] for the intake above the reference of the range from 85.3-104.0 μg/day. OR (95% CI): odds ratio and 95% confidence interval. Min-max: minimum-maximum. Intake estimates were in retinol, μg/day in the present study: (Vitamin A (1 μg) equated (1 μg) of retinol, overall mean ± SD: 102.6 μg/day ± 93.9 (SD: standard deviation)). Retinol Equivalent (RE, one μg) equates one μg of retinol to 6 μg of dietary β-carotene, 12 μg of dietary α-carotene, and 12 μg of dietary β-cryptoxanthin. Retinol Activity Equivalent (RAE, one μg) is equivalent to 1 μg retinol, 12 μg dietary β-carotene, 24 μg of dietary α-carotene, and 24 μg of dietary β-cryptoxanthin. (14)

A similar U-shaped association persisted when excluding cases with early symptoms and matching for sex and age within ± 5 years (Cancer cases, n = 1822; control cases, n = 1822). Both the lowest and highest intakes were associated with an increased cancer risk: OR (95% CI): 1.51 (1.08, 2.09) and OR (95% CI): 2.11 (1.56, 2.86), respectively.

Supplementary Table S2. Vitamin A intake and the risk of cancer by BMI, smoking, alcohol use, blood groups, and sex

Vitamin A intake, mean (min-max), μg/day	Control	Cancer	Crude OR (95%CI)	*P*	Adjusted OR (95%CI)	*P*
**BMI<23, (kg/m²)**						
$P_{\text{\_Trend}}$ [b]			<0.001		<0.001	
18.9 (3.6-29.2)	191	380	2.33 (1.83, 2.96)	<0.001	1.91 (1.46, 2.49)	<0.001
37.1 (29.3-44.3)	189	374	2.31 (1.82, 2.95)	<0.001	1.95 (1.50, 2.53)	<0.001
51.6 (44.4-58.4)	188	362	2.25 (1.77, 2.87)	<0.001	1.78 (1.37, 2.30)	<0.001
64.8 (58.5-71.7)	223	333	1.75 (1.38, 2.22)	<0.001	1.45 (1.13, 1.86)	0.004
78.3 (71.8-85.2)	244	304	1.46 (1.15, 1.85)	0.002	1.30 (1.01, 1.66)	0.039
94.4 (85.3-104.0)	297	254	1.00		1.00	
116.6 (104.1-134.3)	260	294	1.32 (1.04, 1.67)	0.021	1.42 (1.11, 1.82)	0.005
156.1 (134.4-178.2)	269	303	1.32 (1.04, 1.67)	0.021	1.48 (1.16, 1.89)	0.002
312.8 (178.3-704.5)	260	341	1.53 (1.22, 1.94)	<0.001	2.04 (1.59, 2.61)	<0.001
$P_{\text{\_Trend}}$ [c]			0.001		0.001	
**BMI ≥23 (kg/m²)**						
$P_{\text{\_Trend}}$ [b]			<0.001		0.026	
19.1 (3.6-29.2)	62	118	3.38 (2.22, 5.16)	<0.001	2.15 (1.32, 3.50)	0.002
37.8 (29.3-44.3)	68	119	3.11 (2.05, 4.71)	<0.001	2.06 (1.30, 3.27)	0.002
51.9 (44.4-58.4)	92	108	2.09 (1.40, 3.12)	<0.001	1.48 (0.95, 2.28)	0.08
65 (58.5-71.7)	86	109	2.25 (1.50, 3.38)	<0.001	1.69 (1.10, 2.60)	0.018
78.9 (71.8-85.2)	109	93	1.52 (1.02, 2.26)	0.041	1.41 (0.92, 2.15)	0.11
93.3 (85.3-104.0)	128	72	1.00		1.00	
115.7 (104.1-134.3)	119	77	1.15 (0.77, 1.73)	0.50	1.26 (0.82, 1.93)	0.29
154.1 (134.4-178.2)	117	61	0.93 (0.61, 1.42)	0.72	1.23 (0.78, 1.92)	0.37
278.5 (178.3-704.5)	93	56	1.07 (0.69, 1.66)	0.76	1.92 (1.19, 3.08)	0.008
$P_{\text{\_Trend}}$ [c]			0.85		**0.29**	
P for heterogeneity			0.13		0.68	
**Never smoking**						
$P_{\text{\_Trend}}$ [b]			0.001		0.000	
18.6 (3.6-29.2)	178	318	2.55 (1.95, 3.33)	<0.001	1.91 (1.42, 2.57)	<0.001
37.4 (29.3-44.3)	160	309	2.76 (2.10, 3.62)	<0.001	2.24 (1.67, 3.01)	<0.001
51.6 (44.4-58.4)	184	310	2.41 (1.84, 3.14)	<0.001	1.79 (1.35, 2.38)	<0.001
64.8 (58.5-71.7)	196	254	1.85 (1.41, 2.42)	<0.001	1.47 (1.11, 1.95)	0.008
78.3 (71.8-85.2)	205	245	1.71 (1.30, 2.23)	<0.001	1.53 (1.15, 2.02)	0.003
94.4 (85.3-104.0)	247	173	1.00		1.00	
116.1 (104.1-134.3)	216	221	1.46 (1.12, 1.91)	0.006	1.65 (1.24, 2.19)	0.001
155.8 (134.4-178.2)	230	163	1.01 (0.77, 1.34)	0.93	1.22 (0.91, 1.64)	0.18
299.7 (178.3-704.5)	206	197	1.37 (1.04, 1.80)	0.027	2.12 (1.58, 2.85)	<0.001

$P_{\_Trend}$ [c]			0.043		0.016	
**Smokers**						
$P_{\_Trend}$ b			<0.001		0.005	
19.5 (3.6-29.2)	75	180	2.79 (1.98, 3.94)	<0.001	2.10 (1.44, 3.07)	<0.001
36.9 (29.3-44.3)	97	184	2.21 (1.59, 3.06)	<0.001	1.66 (1.17, 2.36)	0.005
51.8 (44.4-58.4)	96	160	1.94 (1.39, 2.70)	<0.001	1.50 (1.06, 2.14)	0.023
65 (58.5-71.7)	113	188	1.94 (1.41, 2.66)	<0.001	1.53 (1.09, 2.13)	0.013
78.7 (71.8-85.2)	148	152	1.19 (0.87, 1.63)	0.26	1.04 (0.75, 1.44)	0.82
93.8 (85.3-104.0)	178	153	1.00		1.00	
116.6 (104.1-134.3)	163	150	1.07 (0.79, 1.46)	0.66	1.12 (0.81, 1.54)	0.49
155.4 (134.4-178.2)	156	201	1.50 (1.11, 2.02)	0.008	1.67 (1.22, 2.29)	0.001
313.2 (178.3-704.5)	147	200	1.58 (1.17, 2.14)	0.003	2.00 (1.45, 2.75)	<0.001
$P_{\_Trend}$ [c]			0.003		0.001	
P for heterogeneity			0.013		0.11	
**No Alcohol use**						
$P_{\_Trend}$ [b]			<0.001		0.001	
18.8 (3.6-29.2)	165	309	2.58 (1.96, 3.39)	<0.001	2.03 (1.50, 2.74)	<0.001
37.1 (29.3-44.3)	160	269	2.32 (1.76, 3.06)	<0.001	1.98 (1.47, 2.67)	<0.001
51.4 (44.4-58.4)	161	240	2.05 (1.55, 2.72)	<0.001	1.59 (1.18, 2.14)	0.002
64.8 (58.5-71.7)	193	197	1.41 (1.06, 1.86)	0.017	1.24 (0.93, 1.66)	0.14
78.4 (71.8-85.2)	204	215	1.45 (1.10, 1.91)	0.008	1.38 (1.04, 1.83)	0.027
94.5 (85.3-104.0)	237	172	1.00		1.00	
116.1 (104.1-134.3)	196	190	1.34 (1.01, 1.77)	0.043	1.41 (1.06, 1.89)	0.019
154.8 (134.4-178.2)	203	174	1.18 (0.89, 1.57)	0.24	1.37 (1.02, 1.84)	0.034
306.8 (178.3-704.5)	187	203	1.50 (1.13, 1.98)	0.005	1.91 (1.42, 2.57)	<0.001
$P_{\_Trend}$ [c]			0.002		0.003	
**Alcohol drinking**						
$P_{\_Trend}$ b			0.003		0.022	
19.2 (3.6-29.2)	88	189	2.62 (1.88, 3.65)	<0.001	1.91 (1.32, 2.77)	0.001
37.4 (29.3-44.3)	97	224	2.82 (2.05, 3.88)	<0.001	2.05 (1.44, 2.91)	<0.001
52 (44.4-58.4)	119	230	2.36 (1.74, 3.21)	<0.001	1.83 (1.31, 2.56)	<0.001
65 (58.5-71.7)	116	245	2.58 (1.90, 3.50)	<0.001	1.78 (1.28, 2.48)	0.001
78.5 (71.8-85.2)	149	182	1.49 (1.10, 2.02)	0.010	1.25 (0.91, 1.73)	0.17
93.7 (85.3-104.0)	188	154	1.00		1.00	
116.6 (104.1-134.3)	183	181	1.21 (0.90, 1.62)	0.21	1.39 (1.01, 1.90)	0.040
156.4 (134.4-178.2)	183	190	1.27 (0.94, 1.70)	0.11	1.53 (1.12, 2.09)	0.008
305.1 (178.3-704.5)	166	194	1.43 (1.06, 1.92)	0.019	2.32 (1.68, 3.20)	<0.001
$P_{\_Trend}$ [c]			0.05		0.015	
P for heterogeneity			0.17		0.09	
**Blood A**						
$P_{\_Trend}$ [b]			0.042		0.21	

19.2 (3.6-29.2)	38	31	3.26 (1.62, 6.58)	0.001	2.88 (1.37, 6.06)	0.005
37.4 (29.3-44.3)	35	33	3.77 (1.87, 7.61)	<0.001	3.32 (1.58, 6.97)	0.001
52 (44.4-58.4)	52	29	2.23 (1.12, 4.44)	0.022	2.24 (1.08, 4.61)	0.029
65 (58.5-71.7)	54	34	2.52 (1.29, 4.93)	0.007	2.47 (1.22, 5.00)	0.012
78.5 (71.8-85.2)	58	29	2.00 (1.01, 3.96)	0.046	2.00 (0.98, 4.09)	0.06
93.8 (85.3-104.0)	72	18	1.00		1.00	
117.3 (104.1-134.3)	61	26	1.70 (0.85, 3.40)	0.13	1.74 (0.84, 3.58)	0.13
156.4 (134.4-178.2)	65	17	1.05 (0.50, 2.20)	0.90	0.99 (0.46, 2.15)	0.98
310.8 (178.3-704.5)	42	41	3.90 (1.99, 7.65)	<0.001	3.55 (1.74, 7.26)	0.001
P_{Trend} [c]			0.015		0.033	
**Blood B**						
P_{Trend} b			0.019		0.11	
19.4 (3.6-29.2)	56	36	2.83 (1.52, 5.29)	0.001	1.75 (0.88, 3.47)	0.10
37.7 (29.3-44.3)	42	27	2.83 (1.45, 5.54)	0.002	1.93 (0.93, 4.00)	0.07
51.4 (44.4-58.4)	61	21	1.52 (0.77, 2.99)	0.22	1.27 (0.62, 2.63)	0.51
65.1 (58.5-71.7)	68	39	2.53 (1.38, 4.64)	0.003	2.35 (1.23, 4.50)	0.010
79 (71.8-85.2)	95	34	1.58 (0.86, 2.89)	0.14	1.48 (0.78, 2.81)	0.23
94.5 (85.3-104.0)	97	22	1.00		1.00	
116.8 (104.1-134.3)	86	35	1.79 (0.98, 3.29)	0.06	1.88 (0.98, 3.61)	0.06
155.4 (134.4-178.2)	91	28	1.36 (0.72, 2.54)	0.34	1.31 (0.67, 2.56)	0.43
289.1 (178.3-704.5)	75	36	2.12 (1.15, 3.90)	0.016	2.09 (1.10, 3.99)	0.025
P_{Trend} [c]			0.005		0.046	
**Blood O**						
P_{Trend} [b]			<0.001		0.044	
18.9 (3.6-29.2)	76	36	2.71 (1.55, 4.74)	<0.001	1.65 (0.91, 3.01)	0.10
37.3 (29.3-44.3)	82	37	2.58 (1.49, 4.49)	0.001	1.99 (1.11, 3.55)	0.020
52 (44.4-58.4)	95	37	2.23 (1.29, 3.85)	0.004	1.62 (0.91, 2.88)	0.10
64.7 (58.5-71.7)	115	38	1.89 (1.10, 3.24)	0.020	1.62 (0.92, 2.84)	0.09
78.4 (71.8-85.2)	127	33	1.49 (0.86, 2.58)	0.15	1.27 (0.72, 2.24)	0.41
94.2 (85.3-104.0)	166	29	1.00		1.00	
115.9 (104.1-134.3)	138	53	2.20 (1.33, 3.65)	0.002	1.88 (1.11, 3.20)	0.019
155.3 (134.4-178.2)	140	42	1.72 (1.02, 2.90)	0.043	1.56 (0.90, 2.69)	0.11
299.4 (178.3-704.5)	98	75	4.38 (2.67, 7.19)	<0.001	3.75 (2.21, 6.38)	<0.001
P_{Trend} [c]			<0.001		0.025	
P for heterogeneity			0.025		0.06	
**Men**						
P_{Trend} [b]			<0.001		0.006	
19.2 (3.6-29.2)	127	280	2.71 (2.06, 3.58)	<0.001	2.10 (1.55, 2.85)	<0.001
37 (29.3-44.3)	154	289	2.31 (1.77, 3.02)	<0.001	1.81 (1.36, 2.42)	<0.001
51.7 (44.4-58.4)	150	266	2.18 (1.67, 2.86)	<0.001	1.74 (1.31, 2.32)	<0.001
65 (58.5-71.7)	166	255	1.89 (1.45, 2.47)	<0.001	1.55 (1.18, 2.06)	0.002

78.6 (71.8-85.2)	206	218	1.30 (1.00, 1.69)	0.049	1.16 (0.88, 1.53)	0.27
93.9 (85.3-104.0)	261	212	1.00		1.00	
116.3 (104.1-134.3)	242	223	1.13 (0.88, 1.47)	0.33	1.24 (0.95, 1.62)	0.11
155.5 (134.4-178.2)	228	251	1.36 (1.05, 1.75)	0.019	1.58 (1.21, 2.06)	0.001
307.6 (178.3-704.5)	223	273	1.51 (1.17, 1.94)	0.001	2.04 (1.56, 2.67)	<0.001
$P_{\_Trend}$ [c]			0.001		0.002	
**Women**						
$P_{\_Trend}$ [b]			0.004		0.001	
18.5 (3.6-29.2)	126	218	2.49 (1.80, 3.44)	<0.001	1.92 (1.34, 2.76)	<0.001
37.5 (29.3-44.3)	103	204	2.85 (2.04, 3.99)	<0.001	2.38 (1.65, 3.44)	<0.001
51.7 (44.4-58.4)	130	204	2.26 (1.63, 3.12)	<0.001	1.63 (1.15, 2.31)	0.006
64.7 (58.5-71.7)	143	187	1.88 (1.36, 2.60)	<0.001	1.35 (0.96, 1.91)	0.08
78.3 (71.8-85.2)	147	179	1.75 (1.27, 2.42)	0.001	1.51 (1.07, 2.13)	0.019
94.5 (85.3-104.0)	164	114	1.00		1.00	
116.4 (104.1-134.3)	137	148	1.55 (1.11, 2.17)	0.010	1.68 (1.18, 2.39)	0.004
155.9 (134.4-178.2)	158	113	1.03 (0.73, 1.45)	0.87	1.19 (0.83, 1.70)	0.34
302.9 (178.3-704.5)	130	124	1.37 (0.97, 1.93)	0.07	2.02 (1.39, 2.91)	<0.001
$P_{\_Trend}$ [c]			0.09		0.011	
P for heterogeneity			0.10		0.44	

[a] Model 1: Adjusted for sex (men and women) (if applicable), age groups (15-39, 40-49, 50-59, 60-69, 70-79, ≥80), highest education level (primary, secondary, high school or higher), blood groups (A, AB, B, O), BMI (kg/m^2, <18.5, 18.5-<23, 23-<25, ≥25), alcohol consumption (yes/no), family history of cancer (yes/no), smoking status (ever/never), history of diabetes (yes/no), coffee drinking (yes/no), total energy intake (kcal/day, nine quantiles), and four periods of data connections. *P_trend* [b] for the intake below the reference of the range from 85.3-104.0 μg/day, *P_trend* [c] for the intake above the reference of the range from 85.3-104.0 μg/day. OR (95%CI): odds ratio and 95% confidence interval. Min-max: minimum-maximum. Intake estimates were in retinol, μg/day in the present study: (Vitamin A (1 μg) equated (1 μg) of retinol, overall mean ± SD: 102.6 μg/day ± 93.9 (SD: standard deviation)). Retinol Equivalent (RE, one μg) equates one μg of retinol to 6 μg of dietary β-carotene, 12 μg of dietary α-carotene, and 12 μg of dietary β-cryptoxanthin. Retinol Activity Equivalent (RAE, one μg) is equivalent to 1 μg retinol, 12 μg dietary β-carotene, 24 μg of dietary α-carotene, and 24 μg of dietary β-cryptoxanthin. (14)

A U-shaped association was observed in the subgroup analyses, including sex, body mass index status, smoking, alcohol consumption, and blood types (A, B, O). **(Supplementary Table S2)**

Supplementary Table S3. Vitamin A intake and the risk of specific cancer sites.

Vitamin A intake, min-max, μg/day	Control	Cancer	Crude OR (95%CI)	*P*	Adjusted OR (95%CI)	*P*
**Esophagus (C15: n=195)**						
$P_{\_Trend}$ [b]			0.11		0.015	
3.6-29.2 μg/day	253	28	3.36 (1.74, 6.50)	<0.001	1.71 (0.82, 3.56)	0.14
29.3-44.3 μg/day	257	24	2.83 (1.44, 5.58)	0.003	1.62 (0.77, 3.41)	0.20
44.4-58.4 μg/day	280	33	3.58 (1.88, 6.81)	<0.001	1.98 (0.98, 4.01)	0.06
58.5-71.7 μg/day	309	24	2.36 (1.20, 4.63)	0.013	1.63 (0.79, 3.38)	0.18
71.8-85.2 μg/day	353	21	1.81 (0.91, 3.60)	0.09	1.38 (0.66, 2.87)	0.38
**85.3-104.0 μg/day**	**425**	**14**	**1.00**		**1.00**	
104.1-134.3 μg/day	379	18	1.44 (0.71, 2.94)	0.31	1.94 (0.91, 4.14)	0.08
134.4-178.2 μg/day	386	20	1.57 (0.78, 3.16)	0.20	2.06 (0.98, 4.34)	0.06
178.3-704.5 μg/day	353	13	1.12 (0.52, 2.41)	0.77	2.71 (1.18, 6.22)	0.019
$P_{\_Trend}$ [c]			0.45		<0.001	
**Stomach (C16: n=1182)**						
$P_{\_Trend}$ [b]			0.002		0.43	
3.6-29.2 μg/day	253	137	2.45 (1.80, 3.32)	<0.001	1.38 (0.99, 1.94)	0.06
29.3-44.3 μg/day	257	136	2.39 (1.76, 3.25)	<0.001	1.70 (1.22, 2.37)	0.002
44.4-58.4 μg/day	280	109	1.76 (1.29, 2.41)	<0.001	1.37 (0.98, 1.91)	0.06
58.5-71.7 μg/day	309	133	1.95 (1.44, 2.63)	<0.001	1.65 (1.20, 2.27)	0.002
71.8-85.2 μg/day	353	120	1.54 (1.13, 2.08)	0.006	1.39 (1.01, 1.91)	0.043
**85.3-104.0 μg/day**	**425**	**94**	**1.00**		**1.00**	
104.1-134.3 μg/day	379	135	1.61 (1.20, 2.17)	0.002	1.46 (1.07, 2.00)	0.017
134.4-178.2 μg/day	386	115	1.35 (0.99, 1.83)	0.06	1.26 (0.91, 1.74)	0.16
178.3-704.5 μg/day	353	203	2.60 (1.96, 3.45)	<0.001	2.21 (1.63, 3.00)	<0.001
$P_{\_Trend}$ [c]			<0.001		<0.001	
**Colon (C18: n=567)**						
$P_{\_Trend}$ [b]			0.011		0.023	
3.6-29.2 μg/day	253	67	1.63 (1.13, 2.36)	0.010	1.40 (0.93, 2.10)	0.10
29.3-44.3 μg/day	257	64	1.53 (1.06, 2.23)	0.025	1.39 (0.94, 2.07)	0.10
44.4-58.4 μg/day	280	62	1.36 (0.94, 1.98)	0.10	1.18 (0.80, 1.74)	0.40
58.5-71.7 μg/day	309	64	1.28 (0.88, 1.85)	0.19	1.15 (0.79, 1.68)	0.47
71.8-85.2 μg/day	353	57	0.99 (0.68, 1.45)	0.97	0.90 (0.61, 1.33)	0.60
**85.3-104.0 μg/day**	**425**	**69**	**1.00**		**1.00**	
104.1-134.3 μg/day	379	56	0.91 (0.62, 1.33)	0.62	0.94 (0.64, 1.39)	0.77
134.4-178.2 μg/day	386	74	1.18 (0.83, 1.69)	0.36	1.28 (0.89, 1.84)	0.18
178.3-704.5 μg/day	353	54	0.94 (0.64, 1.38)	0.76	1.16 (0.78, 1.74)	0.45
$P_{\_Trend}$ [c]			0.83		0.94	

**Rectum (C20: n=482)**						
P_{Trend} [b]			0.059		0.298	
3.6-29.2 µg/day	253	64	2.69 (1.76, 4.11)	<0.001	1.78 (1.12, 2.81)	0.014
29.3-44.3 µg/day	257	60	2.48 (1.62, 3.81)	<0.001	1.93 (1.23, 3.03)	0.004
44.4-58.4 µg/day	280	47	1.78 (1.14, 2.79)	0.011	1.44 (0.91, 2.28)	0.11
58.5-71.7 µg/day	309	44	1.51 (0.96, 2.38)	0.073	1.33 (0.84, 2.11)	0.22
71.8-85.2 µg/day	353	67	2.02 (1.33, 3.06)	0.001	1.80 (1.18, 2.74)	0.007
**85.3-104.0 µg/day**	**425**	**40**	**1.00**		**1.00**	
104.1-134.3 µg/day	379	43	1.21 (0.77, 1.89)	0.41	1.14 (0.72, 1.81)	0.56
134.4-178.2 µg/day	386	57	1.57 (1.02, 2.40)	0.039	1.50 (0.98, 2.32)	0.06
178.3-704.5 µg/day	353	60	1.81 (1.18, 2.76)	0.006	1.70 (1.10, 2.64)	0.017
P_{Trend} [c]			0.18		0.67	
**Lung (C34: n=225)**						
P_{Trend} [b]			0.008		0.004	
3.6-29.2 µg/day	253	40	5.6 (2.88, 10.87)	<0.001	2.08 (1.01, 4.30)	0.047
29.3-44.3 µg/day	257	34	4.69 (2.38, 9.21)	<0.001	2.14 (1.03, 4.45)	0.042
44.4-58.4 µg/day	280	46	5.82 (3.03, 11.18)	<0.001	2.40 (1.19, 4.82)	0.014
58.5-71.7 µg/day	309	37	4.24 (2.18, 8.27)	<0.001	1.91 (0.94, 3.91)	0.07
71.8-85.2 µg/day	353	21	2.11 (1.02, 4.34)	0.043	1.36 (0.64, 2.91)	0.42
**85.3-104.0 µg/day**	**425**	**12**	**1.00**		**1.00**	
104.1-134.3 µg/day	379	15	1.40 (0.65, 3.03)	0.39	1.68 (0.75, 3.79)	0.20
134.4-178.2 µg/day	386	13	1.19 (0.54, 2.65)	0.66	1.55 (0.67, 3.56)	0.30
178.3-704.5 µg/day	353	7	0.70 (0.27, 1.80)	0.46	1.85 (0.68, 4.99)	0.22
P_{Trend} [c]			0.46		0.91	
**Breast (C50: n=281)**						
P_{Trend} [b]			0.023		0.001	
3.6-29.2 µg/day	126	45	4.51 (2.33, 8.71)	<0.001	1.86 (0.86, 4.00)	0.11
29.3-44.3 µg/day	103	47	5.76 (2.97, 11.16)	<0.001	3.63 (1.69, 7.77)	0.001
44.4-58.4 µg/day	130	50	4.85 (2.53, 9.31)	<0.001	2.62 (1.25, 5.50)	0.011
58.5-71.7 µg/day	143	46	4.06 (2.11, 7.81)	<0.001	2.03 (0.96, 4.27)	0.06
71.8-85.2 µg/day	147	26	2.23 (1.11, 4.50)	0.025	1.56 (0.71, 3.44)	0.26
**85.3-104.0 µg/day**	**164**	**13**	**1.00**		**1.00**	
104.1-134.3 µg/day	137	19	1.75 (0.83, 3.67)	0.13	2.06 (0.90, 4.74)	0.08
134.4-178.2 µg/day	158	23	1.84 (0.90, 3.75)	0.09	2.86 (1.28, 6.41)	0.011
178.3-704.5 µg/day	130	12	1.16 (0.51, 2.64)	0.71	3.45 (1.36, 8.71)	0.009
P_{Trend} [c]			0.25		0.002	
**Other cancers (n=826)**						
P_{Trend} [b]			0.026		0.002	
3.6-29.2 µg/day	253	116	1.90 (1.38, 2.63)	<0.001	1.24 (0.86, 1.78)	0.24
29.3-44.3 µg/day	257	125	2.02 (1.47, 2.78)	<0.001	1.44 (1.00, 2.06)	0.047
44.4-58.4 µg/day	280	122	1.81 (1.32, 2.49)	<0.001	1.27 (0.89, 1.80)	0.18

58.5-71.7 μg/day	309	94	1.26 (0.91, 1.76)	0.16	1.10 (0.77, 1.58)	0.60
**71.8-85.2 μg/day**	**353**	**85**	**1.00**		**1.00**	
85.3-104.0 μg/day	425	84	0.82 (0.59, 1.15)	0.24	1.17 (0.82, 1.67)	0.38
104.1-134.3 μg/day	379	84	0.92 (0.66, 1.29)	0.62	1.56 (1.08, 2.24)	0.016
134.4-178.2 μg/day	386	62	0.67 (0.47, 0.95)	0.027	1.19 (0.81, 1.76)	0.37
178.3-704.5 μg/day	353	48	0.56 (0.38, 0.83)	0.003	1.43 (0.94, 2.18)	0.09
$P_{\_Trend}$ [c]			0.001		0.002	

[a] Model 1: Adjusted for sex (men and women), age groups (15-39, 40-49, 50-59, 60-69, 70-79, ≥80), highest education level (primary, secondary, high school or higher), blood groups (A, AB, B, O), BMI (kg/m^2, <18.5, 18.5-<23, 23-<25, ≥25), alcohol consumption (yes/no), family history of cancer (yes/no), smoking status (ever/never), history of diabetes (yes/no), coffee drinking (yes/no), total energy intake (kcal/day, nine quantiles), and four periods of data connections. *P_trend* [b] for the intake below the reference of the range from 85.3-104.0 μg/day, *P_trend* [c] for the intake above the reference of the range from 85.3-104.0 μg/day. OR (95%CI): odds ratio and 95% confidence interval. Min-max: minimum-maximum. Intake estimates were in retinol, μg/day in the present study: (Vitamin A (1 μg) equated (1 μg) of retinol, overall mean ± SD: 102.6 μg/day ± 93.9 (SD: standard deviation)). Retinol Equivalent (RE, one μg) equates one μg of retinol to 6 μg of dietary β-carotene, 12 μg of dietary α-carotene, and 12 μg of dietary β-cryptoxanthin. Retinol Activity Equivalent (RAE, one μg) is equivalent to 1 μg retinol, 12 μg dietary β-carotene, 24 μg of dietary α-carotene, and 24 μg of dietary β-cryptoxanthin. (14)

The lowest and highest quantile consumption was also associated with an increased risk of cancer of the rectum and stomach, but not for the colon. The lowest quantile of consumption increased the risk of lung cancer, with OR (95% CI): 2.08 (1.01, 4.30). **(Supplementary Table S3)**

Supplementary Figure S1. Kernel density estimates for the control cases

The more likely the residuals are normally distributed

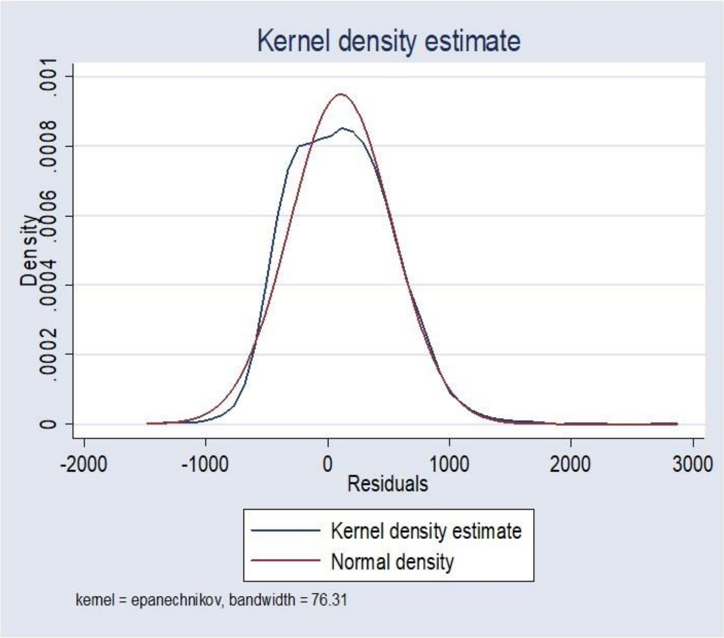

References

1. Ngoan LT, Hung LX, Phu PV, et al. Reproducibility of a semi-quantitative food frequency questionnaire in general populations in North Vietnam. Southeast Asian Journal of Science 2018;6:121-200.
2. Ngoan LT, Khan NC, Mai le B, et al. Development of a semi-quantitative food frequency questionnaire for dietary studies--focus on vitamin C intake. Asian Pac J Cancer Prev 2008;9:427-32.
3. Tomova GD, Arnold KF, Gilthorpe MS, Tennant PWG. Adjustment for energy intake in nutritional research: a causal inference perspective. Am J Clin Nutr 2022;115:189-198.
4. Ghyselinck NB, Duester G. Retinoic acid signaling pathways. Development 2019;146.
5. Kersten S, Gronemeyer H, Noy N. The DNA binding pattern of the retinoid X receptor is regulated by ligand-dependent modulation of its oligomeric state. J Biol Chem 1997;272:12771-7.
6. Heyman RA, Mangelsdorf DJ, Dyck JA, et al. 9-cis retinoic acid is a high-affinity ligand for the retinoid X receptor. Cell 1992;68:397-406.
7. Dillard AC, Lane MA. Retinol Increases beta-catenin-RXRalpha binding leading to the increased proteasomal degradation of beta-catenin and RXRalpha. Nutr Cancer 2008;60:97-108.
8. Park EY, Dillard A, Williams EA, Wilder ET, Pepper MR, Lane MA. Retinol inhibits the growth of all-trans-retinoic acid-sensitive and all-trans-retinoic acid-resistant colon cancer cells through a retinoic acid receptor-independent mechanism. Cancer Res 2005;65:9923-33.
9. Schug TT, Berry DC, Shaw NS, Travis SN, Noy N. Opposing effects of retinoic acid on cell growth result from alternate activation of two different nuclear receptors. Cell 2007;129:723-33.
10. Paolini M, Abdel-Rahman SZ, Sapone A, et al. Beta-carotene: a cancer chemopreventive agent or a co-carcinogen? Mutat Res 2003;543:195-200.
11. Arab L, Steck-Scott S, Bowen P. Participation of lycopene and beta-carotene in carcinogenesis: defenders, aggressors, or passive bystanders? Epidemiol Rev 2001;23:211-30.
12. Murata M, Kawanishi S. Oxidative DNA damage by vitamin A and its derivative via superoxide generation. J Biol Chem 2000;275:2003-8.
13. Cha JH, Yu QM, Seo JS. Vitamin A supplementation modifies the antioxidant system in rats. Nutr Res Pract 2016;10:26-32.
14. Ministry of Health, National Institute of Nutrition. Vietnamese Food Composition Table. Hanoi city: Medical Publishing House, 2007.
